# Preparation of Modified Beeswax and Its Influence on the Surface Properties of Compressed Poplar Wood

**DOI:** 10.3390/ma9040230

**Published:** 2016-03-25

**Authors:** Lili Ren, Yingchun Cai, Limin Ren, Hong Yang

**Affiliations:** Key Laboratory of Bio-based Material Science and Technology (Ministry of Education), National Natural Science Foundation of China (31270595), Northeast Forestry University, Harbin 150040, China; renlili@nefu.edu.cn (L.R.); renlimin1976@126.com (L.R.); yanghong@nefu.edu.cn (H.Y.)

**Keywords:** beeswax, chitosan, nano-TiO_2_, dye, hot waxing, surface properties of compressed wood

## Abstract

Beeswax was modified through the direct blending of nano-TiO_2_, chitosan, acid dyes, and neutral dyes. With the varied modified beeswax, hot waxing was conducted on compressed poplar wood. Treated wood surfaces were characterized with scanning electron microscopy and thermogravimetric analysis. Results show that the C, O, N, Au, S, and Ti elements in modified beeswax successfully penetrated into pits of compressed poplar wood, imparting rich colors to the wood surface. The blending of 10% (mass fraction) beeswax, 0.2% chitosan, and 0.05% neutral red was the best treatment for mulation for improving the in-surface staining uniformity, durability, surface gloss, contact angle, and texture conspicuity of compressed poplar.

## 1. Introduction

Beeswax is a widely used protective material in traditional Chinese wooden architecture and hardwood furniture. During the hot waxing of hardwoods, problems such as discoloration, poor durability, and high cost often occur. China is a country lacking valuable timber resources but is rich in fast-growing wood resources. How does one effectively use fast-growing wood resources, reduce costs, and improve the surface properties of wood? One possible solution is to treat the wood surface with hot modified beeswax.

In recent years, scholars have studied the engineering process of wax modification and traditional waxing, and factors affecting the quality of waxing [1,2,3]. Krause *et al.* studied the preparation process of lignite wax and improved antibacterial and anticorrosion properties of the wax on wood surfaces [4]. In the synthesis of a new environmentally friendly wax for wood, Li *et al.* directly mixed linseed oil, tung oil, and other natural extracts at a temperature of 130 °C and determined the optimum ratio of the wax by testing hardness, appearance, and the color of products [5]. Scholz *et al.*'s study indicated that amide wax imparts excellent termite resistance to wood [6]. Bonaduce used mass spectrometry and gas chromatography to describe characteristics of the beeswax in art works [7]. Donaldson studied the deformation degree of the tracheid of compressed wood and analyzed the microstructural changes in compressed wood after hot waxing with confocal fluorescence imaging and spectroscopy [8,9,10,11]. Guo conducted research on the durability of Burmese rosewood to ultraviolet light after hot waxing by nano-TiO_2_-modified hydrophobic beeswax [12]. Evans studied the effectiveness of wax and oil emulsion additives, aimed to improve the performance of preservative-treated wood [13]. Levi studied the distribution and effectiveness in *Pinus sp.* of a water-repellent additive for water-borne wood preservatives [14]. However, the aforementioned research did not focus on the description of the preparation process of the effect of modified beeswax and influence of hot waxing treatment on wood properties.

Hence, this study used compressed poplar wood as a base material, and conducted hot waxing on it using modified beeswax synthesized by nano-TiO_2_, chitosan, acid dyes, and neutral dyes at different proportions. Then, taking thermal stability, permeability, colorimetric parameters, hydrophobicity, gloss, and texture as evaluation parameters, the poplar wood specimens after treatment were tested and compared in order to provide theoretical evidence for improving the surface properties of compressed poplar wood with modified beeswax. We hypothesized that the direct mixing of 0.05% neutral dye and 0.2% chitosan into 10% beeswax can produce the greatest improvement for the surface performance of compressed poplar wood after hot waxing. Thus, low-cost, efficient, and high value-added products of the modified beeswax-treated compressed poplar wood could be obtained.

## 2. Materials and Methods 

### 2.1. Materials

The compressed wood selected for testing was rotary-cut veneer from transversely compressed *Populus ussuriensis* Kom from Heilongjiang, with a size of 100 mm × 100 mm × 12 mm. The material for hot waxing was yellow beeswax from Sichuan. Modifying materials (chitosan, nano- TiO_2_, acid red, and neutral red) were purchased from Aladdin Reagent.

### 2.2. Test Equipment

A scanning electron microscope (SEM, FEI, Hillsboro, OR, USA), a handheld spectrophotometer, FLUKO-FA25 homogenizer (10,000 r/min), a Thermal Gravimetric Analyzer (TGA, DuPont), a LA2800S scanner, a video-based optical contact angle measuring system OCA20, and a KGZ-IB portable gloss meter (Tianjin Keqi High and New Technology Corporation, Tianjin, China) were employed.

### 2.3. Experimental Methods

Preparation of modified beeswax: Beeswax was added to a 100-mL beaker and melted in a 80-°C water bath. Chitosan, nano-TiO_2_, and dyes were added to the melted beeswax and were stirred in a high-speed FLUKO-FA25 homogenizer (10,000 r/min) at a speed of 10,000 r/min for 1 h to achieve uniform dispersion of chitosan, nano-TiO_2_, and dye particles in the beeswax.

Hot waxing of poplar wood specimen with modified beeswax: The waxing level was 50 g/m^2^, the wax stripping time was 6 minutes, and a baking temperature of 90 °C was used. The melted modified beeswax was dotted with a brush on the buffed compressed wood surface. Then, the wood surface was baked at 150–250 °C with a hot air gun (LEISTER, Kaegiswil, Switzerland) thermal spray for 3 min, followed by low-temperature baking for 30 s to promote the penetration of beeswax in wood pores. After solidification of beeswax, the floating (poorly absorbed) wax was removed from the wood surface using a special shovel and subsequently wiped with a clean cotton cloth.

### 2.4. Performance Test

Wood specimens were subject to hot waxing of mixed beeswax. According to differences in modified materials and the results of previous studies [15], the mass fractions of beeswax, dyes, nano-TiO_2_ and chitosan were 10%, 0.05% 0.2%, and 0.2%, respectively. The corresponding wood specimens treated with these blends are referred to as A1 = beeswax + acid red, A2 = beeswax + acid red + nano-TiO_2_, A3 = beeswax + acid red + chitosan, B1 = beeswax + neutral red, B2 = beeswax + neutral red + nano-TiO_2_, and B3 = beeswax + neutral red + chitosan, successively.

Modified beeswax’s heat resistance test: For the waxed wood specimens A1, A2, A3, B1, B2, and B3 TGA analysis, the measurement range was from room temperature to 500 °C, with a heating rate of 10 °C/min in air. The mass of each sample was 5 mg. Using the thermal decomposition curve of modified beeswax, we compared the thermal stability of six modified materials.

Permeability test: The waxed wood specimens A1, A2, A3, B1, B2, and B3 were cut with a blade into 5 mm × 5 mm × 1 mm specimens, and in this process the cutting edge of the blades was only used once. A scanning electron microscope was used to analyze the microstructure of six kinds of modified beeswax on the surface of compressed poplar wood.

Chromaticity detection: For the waxed wood specimens A1, A2, A3, B1, B2, and B3, an aging test was conducted respectively for 60 h in total using a XXL+ xenon lamp aging chamber. Wood color changes were quantified by the standard colorimetric parameters (brightness L*, red-green chromaticity index a*, yellow-blue chromaticity index b*) recommended by the International Commission on Illumination (CIE). Using a handheld spectrophotometer, those designated test surfaces were detected every 12 h. In addition, we calculated the change of each color parameter before and after UV radiation, *i.e.* ∆L* (brightness difference), ∆a* (red-green chromaticity difference), and ∆E* (color value). Three points were taken for the test surface of each specimen, and the average of these three points was calculated to represent the wood color of specimens. The calculation Equation (1) is as follows:

(1) $$∆E^{*}={\left[{\left(∆L^{*}\right)}^{2}+{\left(∆a^{*}\right)}^{2}+{\left(∆b^{*}\right)}^{2}\right]}^{1/2}$$

Detection of surface hydrophobicity: A video-based optical contact angle measuring system OCA 20 was used to measure the advancing contact angle of the surface of A1, A2, A3, B1, B2, and B3 specimens at different aging stages [16]. The liquid used was distilled water, and the volume of the measuring droplet was 5 μL. The final result was the average from three different positions randomly selected for each specimen.

Detection of surface gloss: A KGZ-IB portable gloss meter (Tianjin Keqi High and New Technology Corporation, Tianjin, China) was used to measure the surface gloss of A1, A2, A3, B1, B2, and B3 specimens, with three detecting points for each specimen. To ensure data accuracy, the mirror gloss meter was calibrated with the value of 25.5°, calibration was performed after the detection on each point. Gloss values are the average of three detecting points.

Test of surface texture features: Wood surface of A1, A2, A3, B1, B2, and B3 specimens were scanned respectively with (LA2800) a scanner at different aging stages. The obtained images were subject to multi-layer decomposition using MATLAB texture program and wavelet analysis to extract surface texture features. The abbreviation LL represents the low-frequency component in orthogonal wavelet decomposition, and LH, HL, and HH represent the high-frequency component of the horizontal edge, vertical edge, and diagonal edge, respectively.

## 3. Results and Analysis

### 3.1. Heat Resistance of Modified Beeswax

**Figure 1:**
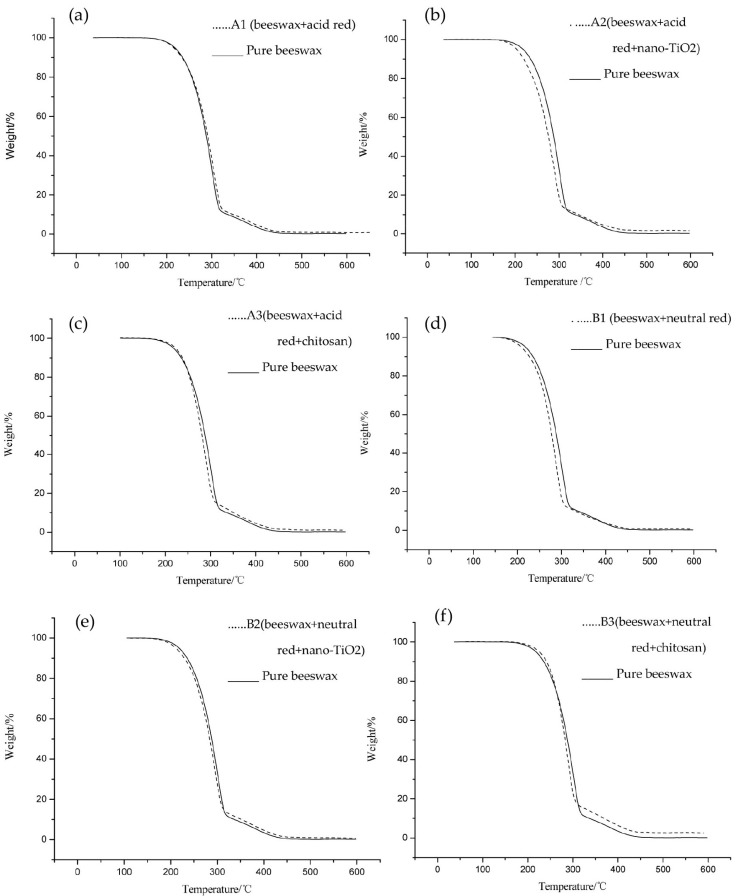
Modified TG wax. (**a**) A1 = beeswax + acid red; (**b**) A2 = beeswax + acid red + nano-TiO_2_; (**c**) A3 = beeswax + acid red + chitosan; (**d**) B1 = beeswax + neutral red; (**e**) B2 = beeswax + neutral red + nano-TiO_2_; (**f**) B3 = beeswax + neutral red + chitosan.

Figure 1 shows the thermogravimetric (TG) curves of pure beeswax and six kinds of modified beeswax. It can be seen that with the increase of temperature, there was major degradation of both pure and modified beeswax. Pure beeswax degraded and lost weight mainly in the range of 155.9–465.2 °C. The B3 = beeswax + neutral red + chitosan, range was widened through the addition of chitosan to 168.9 °C–477.3 °C. The initial degradation temperature and the maximum weight-losing temperature increased from 155.9 °C and 465.2 °C of the pure beeswax to 168.9 °C and 477.3 °C, respectively. The graph shows that the inclusion of chitosan improved the thermal stability of the beeswax. It can be seen from the TG curves that, after thermal degradation, the chitosan-modified beeswax had more residues than the pure wax control. Analysis revealed that the main components of the pure beeswax are esters, free acids, free alcohols, and hydrocarbons, and the residue after pyrolysis was about 0.3% of its original weight. The B3 (beeswax + neutral red + chitosan) ratio was raised to 2.15% after the modification through chitosan [17].

### 3.2. Surface Energy Spectrum of Modified Beeswax-Treated Compressed Poplar Wood

**Figure 2:**
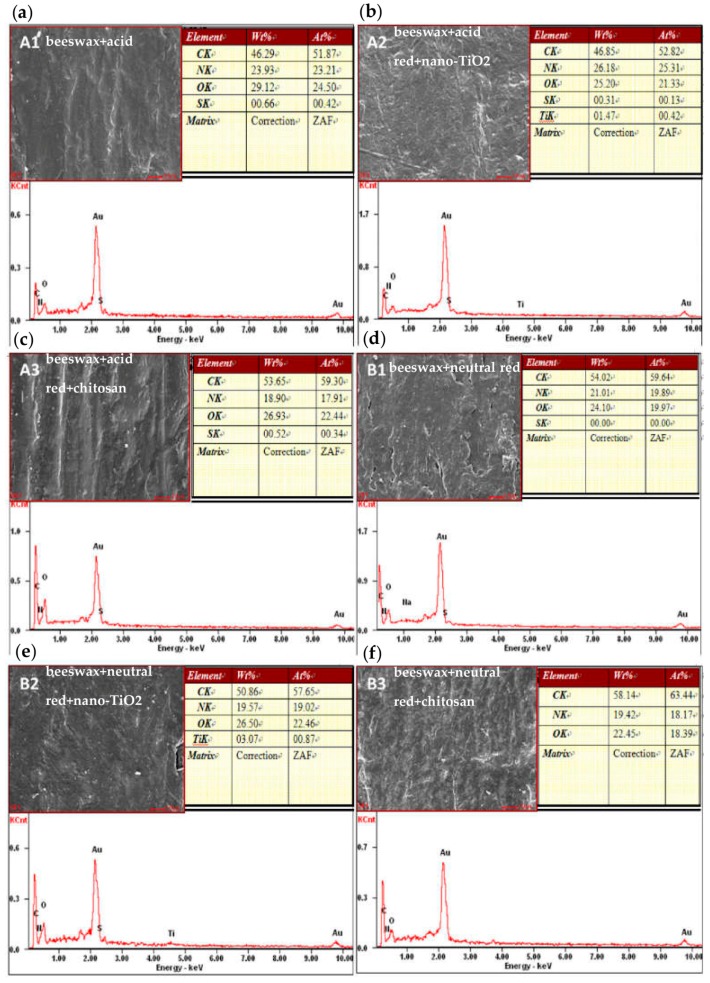
The modified wax compressed poplar X-ray surface energy spectra. (**a**) A1 = beeswax + acid red; (**b**) A2 = beeswax + acid red + nano-TiO_2_; (**c**) A3 = beeswax + acid red + chitosan; (**d**) B1 = beeswax + neutral red; (**e**) B2 = beeswax + neutral red + nano-TiO_2_; (**f**) B3 = beeswax + neutral red + chitosan.

In Figure 2, C, O, N, Au, and S elements can be seen clearly in Figure 2a (beeswax + acid red) and 3a (beeswax + acid red + chitosan). Besides these five elements, Ti can also be seen in Figure 2b (beeswax + acid red + nano-TiO_2_). As for Figure 2d (beeswax + neutral red) and Figure 2f (beeswax + neutral red + chitosan), C, O, N, and Au can be clearly seen, and from Figure 2e (beeswax + neutral red + nano-TiO_2_) we observed five elements: C, O, N, Au, and Ti. Therein, C mainly came from the wood; Au came from the coating process during the sample preparation for electron microscopy; O partly originated from the wood itself and partly from the hot waxing; N came partly from chitosan and partly from acidic red and neutral red dyes; S was absorbed from the acid red dye; and Ti was derived from the nano-TiO_2_ added for modification and can be observed in the thin wax layer. This suggests that hot waxing treatment successfully attached acid red, neutral red, nano-TiO_2_, and chitosan to the wood surface [18].

### 3.3. Permeability of Modified Beeswax on the Surface of Compressed Poplar Wood

**Figure 3:**
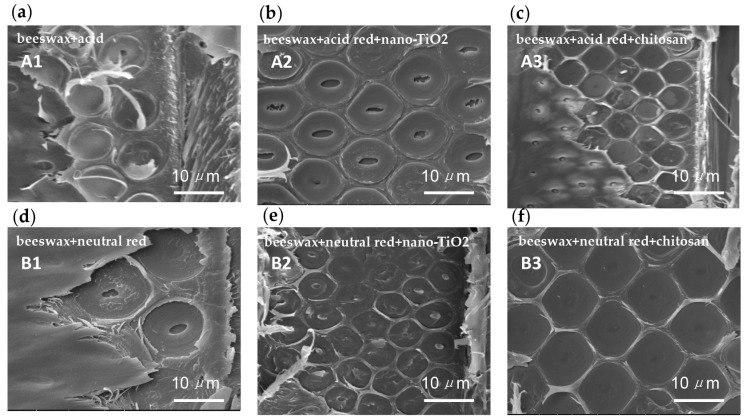
The modified wax on the compressed poplar surfaces. (**a**) A1 = beeswax + acid red; (**b**) A2 = beeswax + acid red + nano-TiO_2_; (**c**) A3 = beeswax + acid red + chitosan; (**d**) B1 = beeswax + neutral red; (**e**) B2 = beeswax + neutral red + nano-TiO_2_; (**f**) B3 = beeswax + neutral red + chitosan.

In Figure 3, it can be clearly seen that samples A1 = beeswax + acid red, A3 = beeswax + acid red + chitosan, B1 = beeswax + neutral red, B2 = beeswax + neutral red + nano-TiO_2_, and B3 = beeswax + neutral red + chitosan, have pits on the radial section of compressed poplar wood that are filled with modified beeswax. This indicates that hot waxing treatment has successfully deposited neutral red, acid red, Nano-TiO_2_, and chitosan at wood surfaces. However, some pits in A2 = beeswax + acid red + nano-TiO_2_, were not filled, indicating non-uniform distribution of the modified beeswax directly mixed with acidic red and nano-TiO_2_ into the compressed poplar wood. According to the fully filled pits of B3 = beeswax + neutral red + chitosan, the penetration of the beeswax modified by neutral red and chitosan was optimum.

### 3.4. Surface Colorimetric Parameters of Modified Beeswax-Treated Compressed Poplar Wood

**Figure 4:**
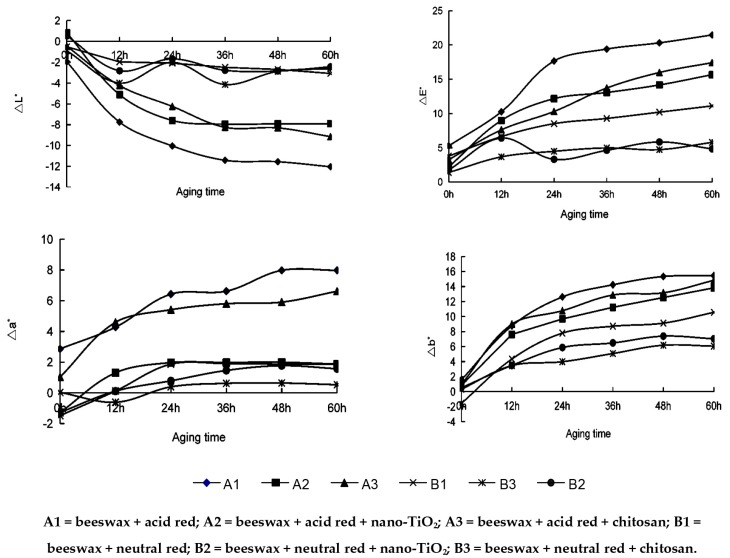
The colour change (∆L* =brightness difference, ∆a* = red-green chromaticity difference, ∆b* = yellow-blue chromaticity index, ∆E* = color value) of modified wax-treated compressed poplar exposed to UV light.

**Figure 5:**
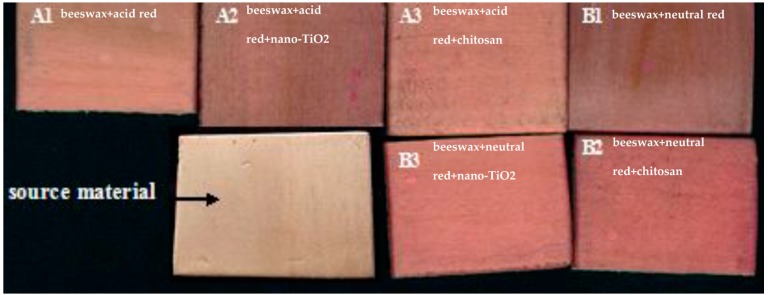
The color change of the modified wax specimen and material surface after 60 h UV-irradiation.

In Figure 4, the ∆L* of A1 = beeswax + acid red, A2 = beeswax + acid red + nano-TiO_2_, A3 = beeswax + acid red + chitosan, B1 = beeswax + neutral red, B2 = beeswax + neutral red + nano-TiO_2_, and B3 = beeswax + neutral red + chitosan, was −12.038, −7.9, −9.146, −3.08, −2.43, and −2.62, respectively. A1 = beeswax + acid red, shows the largest curve amplitude, and the curve is at the bottommost location, indicating the maximum ∆L*. The B2 = beeswax + neutral red + nano-TiO_2_, curve is located in the middle, indicating the minimum ∆L*. B3 = beeswax + neutral red + chitosan and B2 = beeswax + neutral red + nano-TiO_2_, are the most stable among ∆a* curves, and their ∆a* values were −2.62 and −2.436, respectively. In terms of ∆b* curve, A1 has the largest change, while B3 = beeswax + neutral red + chitosan and B2 = beeswax + neutral red + nano-TiO_2_ changed the least. In the ∆E* curve, the color difference of B2 = beeswax + neutral red + nano-TiO_2_ was 4.838, and that of B3 = beeswax + neutral red + chitosan was 5.792, about 20% higher than the former. In Figure 5, after 60h UV-irradiation, there is no big change for the six kinds of modified beeswax wax material and pure beeswax wax. This suggests that modified wax has good aging resistance, as the color change of B2 = beeswax + neutral red + nano-TiO_2_ and B3 = beeswax + neutral red + chitosan was minimal.

In summary, the wood specimens treated by the beeswax modified with 0.2% nano-TiO_2_ and 0.05% neutral red dye had the smallest change in colorimetric parameters. This indicates that this modification can provide short-term (60 h) protection to wood against the UV and inhibit light-induced discoloration of the wood [19].

### 3.5. Surface Hydrophobicity of Modified Beeswax-Treated Compressed Poplar Wood

**Figure 6:**
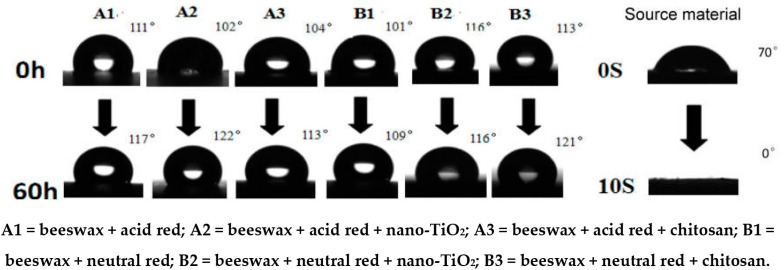
The modified wax specimen and material surface contact angle changes.

It can be seen in Figure 6 that the initial contact angles of all the wood specimens subject to hot waxing were over 100°, reaching a high level of hydrophobicity, while the contact angle of the unwaxed base material was 70°, lacking hydrophobicity. The contact angles of specimen B2 = beeswax + neutral red + nano-TiO_2_ and B3 =beeswax + neutral red + chitosan, after UV exposure for 60 h were 116° and 121°, higher by 4°–11° compared with B1 = beeswax + neutral red, B2 = beeswax + neutral red + nano-TiO_2_, and B3 = beeswax + neutral red + chitosan. These results indicate that the B2 = beeswax + neutral red + nano-TiO_2_ and B3 = beeswax + neutral red + chitosan, specimens slightly improved surface hydrophobicity. The contact angles of A1 = beeswax + acid red, A2 = beeswax + acid red + nano-TiO_2_, A3 = beeswax + acid red + chitosan, and B1 = beeswax + neutral red also remained over 100°. Their contact angles reduced by about 1–1.05 times compared with B2 = beeswax + neutral red + nano-TiO_2_ and by 1–1.1 times compared with B3 = beeswax + neutral red + chitosan.

In summary, the wax will go into wood pores and form a thin layer at the wood surface, effectively improving the hydrophobicity of wood surfaces. The application of acid dye, neutral dye, chitosan, and nano-TiO_2_ can reduce UV damage to the surface wax layer. Since the contact angle of B3 = beeswax + neutral red + chitosan had the smallest reduction, the wood hydrophobicity was strongest after the treatment of the beeswax was modified by 0.2% chitosan and 0.05% neutral red dye.

### 3.6. Influence of Hot Waxing of Modified Beeswax on the Surface Gloss of Compressed Poplar Wood

**Figure 7:**
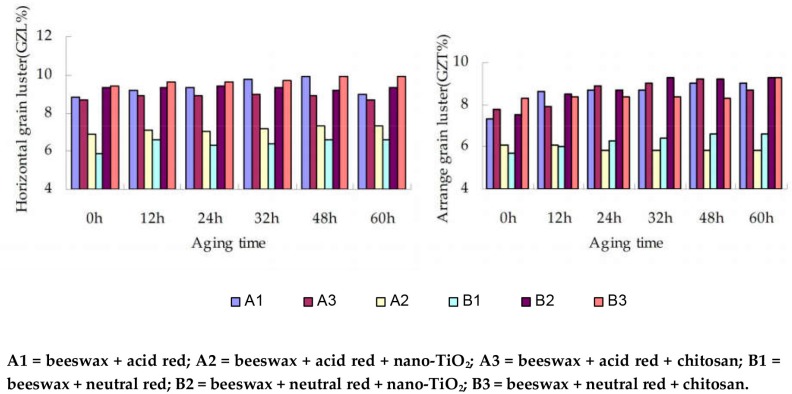
The modified wax compressed poplar grain and grain gloss gloss.

As can be seen in Figure 7, the gloss values of A2, A3, and B1 along and across the grain were all lower than those of A1, B2, and B3. Therein, the gloss value of B3 along the grain was 1.03% and 1% higher than those of A1 and B2, respectively. Additionally, the gloss value of B3 across the grain was also higher by 1.03% and 1.1% respectively compared with the latter two.

In summary, the impact of short-term exposure to UV radiation on the surface gloss of wood is generally small. The gloss values of B2 = beeswax + neutral red + nano-TiO_2_ were slightly lower than those of B3 = beeswax + neutral red + chitosan. This is possibly because the addition of nano-TiO_2_ and acid dye resulted in an increased roughness of the wax layer, and UV light would scatter at the rough surface to form a matting effect, thereby reducing the surface gloss (of B2). The surface gloss of B3 = beeswax + neutral red + chitosan was the closest to the +++TL, *i.e.* the modification by 0.2% chitosan and 0.2% neutral red dye brought about the best wood surface gloss.

### 3.7. Surface Texture of Modified Beeswax-Treated Compressed Poplar Wood

**Figure 8:**
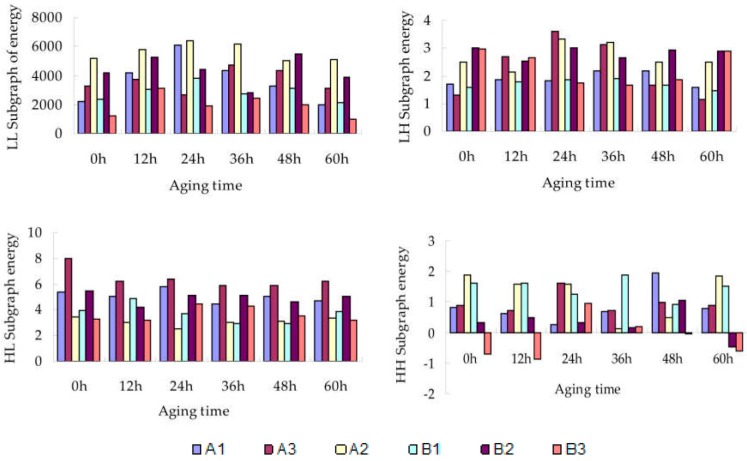
Modified wax on the compressed poplar LL, LH, HL, and HH subgraph energy value changes.

It is clearly understood from wavelet analysis that as ELL (the low frequency LL subgraph of wavelet analysis on the second scale) reduces and as EHL (the medium-high frequency HL subgraph of wavelet analysis on the second scale of vertical edge), ELH (the medium-high frequency LH subgraph of wavelet analysis on the second scale of horizontal edge), and EHH (the high frequency HH subgraph of wavelet analysis on the second scale of diagonal edge) increased, the wood grain became increasingly coarse and deep, indicating that the surface texture of modified beeswax-treated compressed poplar wood became more pronounced In Figure 8, with the increase of irradiation time, the energy-value curves of specimens in the four subgraphs at 12–48 h have gone up or down. However, in general, all curves show a declining trend, indicating that the texture of the specimens became less conspicuous possibly because of the UV-induced aging of the wax layer. After 48 h of UV irradiation, B3 = beeswax + neutral red + chitosan, had the largest decline of 250.1 in subgraph LL, while B1 had the smallest decline of 10.4, indicating a minimum change in B1 = beeswax + neutral red. B3 = beeswax + neutral red + chitosan in subgraph LH had the lowest decline, being only 0.1. Its decline was also the smallest in subgraph HL and HH. This indicates that B3 = beeswax + neutral red + chitosan, treated by the beeswax modified by 0.5% chitosan and 0.2% neutral red dye, maintained its surface texture to a greater extent than the other specimens.

## 4. Conclusions

In conclusion, a mixture of 10% beeswax, 0.05% neutral red, and 0.2% chitosan was the most effective treatment. The direct mixing of 0.05% neutral dye and 0.2% chitosan into 10% beeswax can produce the greatest improvement for the surface performance of compressed poplar wood after hot waxing. As a result of treatment with such modified beeswax, the poplar wood can maintain its color and physical and mechanical properties, including dimensional stability, and it can be used in both dry and damp environments. This provides a high-quality material for furniture and woodcarving production at a lower cost and simultaneously improves the use value of broad-leaved fast-growing species.
